# Adult mosquito predation and potential impact on the sterile insect technique

**DOI:** 10.1038/s41598-022-06565-1

**Published:** 2022-02-15

**Authors:** Nanwintoum Séverin Bimbilé Somda, Hamidou Maïga, Wadaka Mamai, Thierno Bakhoum, Thomas Wallner, Serge Bèwadéyir Poda, Hanano Yamada, Jérémy Bouyer

**Affiliations:** 1 Insect Pest Control Laboratory, Joint FAO/IAEA Centre of Nuclear Techniques in Food and Agriculture, International Atomic Energy Agency, 1400 Vienna, Austria; 2 Institut de Recherche en Sciences de la Santé/Direction Régionale de l’Ouest (IRSS/DRO), 01, BP 545, Bobo-Dioulasso, Burkina Faso; 3 Unité de Formation et de Recherche en Sciences et Technologies (UFR/ST), Université Norbert ZONGO (UNZ), BP 376, Koudougou, Burkina Faso; 4 Institut de Recherche Agricole pour le Développement (IRAD), BP 2123, Yaoundé-Messa, Cameroon; 5 ASTRE, CIRAD, INRAE, Université de Montpellier, Montpellier, France

**Keywords:** Biological techniques, Biotechnology, Ecology

## Abstract

The sterile insect technique is a promising environmentally friendly method for mosquito control. This technique involves releasing laboratory-produced sterile males into a target field site, and its effectiveness may be affected by the extent of adult mosquito predation. Sterile males undergo several treatments. Therefore, it is vital to understand which treatments are essential in minimizing risks to predation once released. The present study investigates the predation propensity of four mantis species (*Phyllocrania paradoxa, Hymenopus coronatus, Blepharopsis mendica, Deroplatys desiccata*) and two gecko species (*Phelsuma standingi, P. laticauda)* on adult *Aedes aegypti*, *Ae. albopictus* and *Anopheles arabiensis* mosquitoes in a laboratory setting. First, any inherent predation preferences regarding mosquito species and sex were evaluated. Subsequently, the effects of chilling, marking, and irradiation, on predation rates were assessed. The selected predators effectively preyed on all mosquito species regardless of the treatment. Predation propensity varied over days for the same individuals and between predator individuals. Overall, there was no impact of laboratory treatments of sterile males on the relative risk of predation by the test predators, unless purposely exposed to double the required sterilizing irradiation dose. Further investigations on standardized predation trials may lead to additional quality control tools for irradiated mosquitoes.

## Introduction

Mosquito-borne diseases such as malaria and dengue are a significant threat to global health. Malaria, a parasitic infection transmitted by Anopheline mosquitoes, is responsible for more than 409,000 deaths in 2019^1^ while dengue fever, a viral infection transmitted by *Aedes* mosquitoes, causes around 40,000 deaths every year^2^. With the absence of reliable vaccines for effective prevention, the management of the main vectors appears to be the best strategy of control^3^. Intervention efforts such as environmental sanitation, indoor residual spray, and long-lasting insecticide-treated bed nets have contributed to limiting the incidence of these diseases in the last decades^3^. However, their burden on public health is still substantial. The weakness of the available control strategies is partly due to the lack of comprehensive understanding of biological interactions, including co-evolution mechanisms between mosquito vectors, pathogens and environment, and the role of mosquito vectors in the food chain of living organisms^4,5^. Therefore, a good knowledge of such interactions in general or regarding a specific control strategy would contribute to developing more effective alternative or complementary vector control methods including biological and genetic techniques.

Natural enemies are well known in biocontrol for their beneficial actions of reducing the density of insect pests^6^. Early mosquito control approaches included the use of natural predators. Mainly, predators of the mosquito aquatic life stages were considered^7–9^. Predators with high predation rates on mosquito larvae and pupae include fish^10,11^, odonate young instars^12^, other mosquito species^13^, amphibians^14^, and copepods^15^. Although attention was mainly given to mosquito pre-imago stage predation, recent studies demonstrated that predators of adult mosquitoes, such as spiders and geckos, have a high potential to reduce mosquito populations^16–20^. Adult mosquito predators also include bats^21^, dragon flies^22^, frogs^23^, birds^24^. More broadly, adult mosquitoes may be an essential part of the food resources of terrestrial predators of arthropods such as mantises, known to prey on small flying insects^25,26^. Therefore, investigating adult mosquito predation would offer more biological control options and is also vital to define its potential interactions with genetic control.

Genetic control techniques, such as the sterile insect technique (SIT), are promising tools as components of area-wide integrated management of mosquitoes. However, these techniques, involve releasing laboratory-produced (sterile) mosquitoes into a target field site, and their effectiveness may be affected by the extent of adult mosquito predation. The SIT is an environment-friendly, species-specific technique releases sterile male insects into a target area which will compete with their wild counterparts to mate with wild females, resulting in a decline of the target insect pest population^27^. Its success relies on the capacity of sterile males to mate with wild females and therefore requires a sufficient number of sterile males of good quality to compete with wild males^28,29^. This requires mastering all components of the technique, from the laboratory to the field, that can critically affect the quality of the produced mosquito. *Aedes aegypti, Ae. albopictus* and *Anopheles arabiensis* are among the species currently targeted by SIT programmes^30^. Significant advances have been made (and are being optimised) in developing SIT package against these mosquitoes, including colonisation, mosquito mass-rearing^31–36^, sex-separation^37–40^, handling^41^, radiation^42,43^, quality control^44,45^, and release^46^. In addition, field investigations such as the ecology of reproduction of mosquitoes^47^ and the sterile male behaviour^48,49^ have been carried out to determine the technique’s feasibility. Recent pilot trials on *Aedes* mosquitoes showed promising results^46,50^. However, much remains to be discovered about biological interactions and the fate of released males in the wild, for instance, the risk of predation by abundant predators. It has been reported that colonisation and mass rearing conditions adversely affect the ability of males of the Mexican fruit fly, *Anastrepha ludens,* to escape spider predation^51^. In addition, Rathnayake et al*.*^52^ found that sterile and fertile males of the Queensland fruit fly, *Bactrocera tryoni*, were similarly vulnerable to predation. Both groups were more prone to predation than females. A good understanding of the impact of adult mosquito predation on laboratory-produced males would therefore contribute to defining the SIT requirements better, especially the quality and the number of mosquitoes to release in a target area.

The present study aims to determine whether the dead leaf mantis species, *Phyllocrania paradoxa, Hymenopus coronatus, Blepharopsis mendica, Deroplatys desiccata* and the day geckos, *Phelsuma standingi,* and *P. laticauda,* prey on adult mosquitoes, *Ae. aegypti, Ae. albopictus* and *An. arabiensis* under laboratory settings. Furthermore, the study assessed mosquito chilling, marking and irradiation on their vulnerability to predation. Finally, the predators’ preferences between mosquito species and sexes were evaluated. The predators were selected based on the following hypotheses: they share their distribution areas with at least one of the assessed mosquito species; the mantises are leaf-flower mantis species that eat pollinators and may have interactions with mosquitoes. The selected gecko species are diurnal species which are more likely to interact with *Aedes* species, currently the main targets of SIT programs.

## Results

### Mantis predation propensity on *Aedes* and *Anopheles* mosquitoes

#### Dynamics of mantis predation on *Aedes* mosquitoes

**Figure 1:**
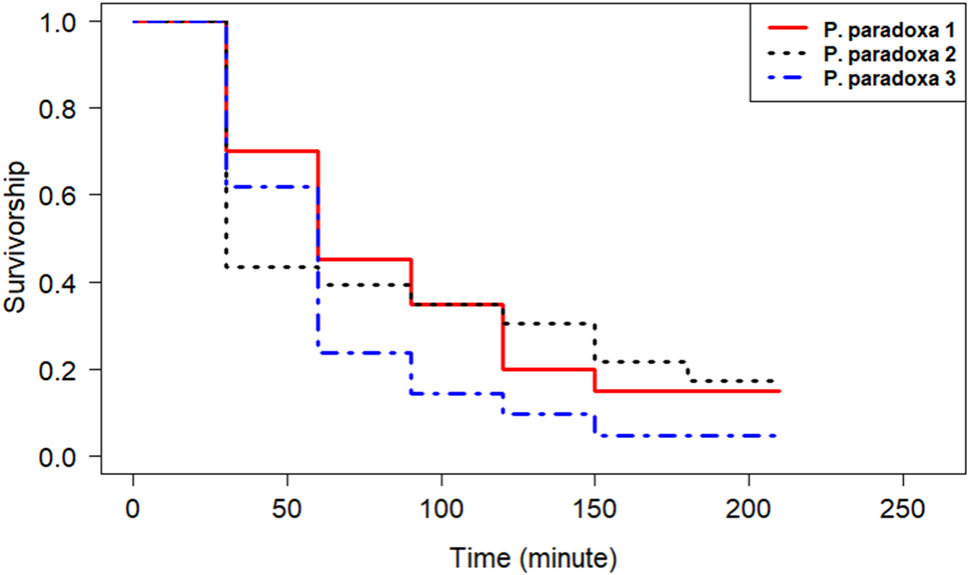
Dynamics of *Phyllocrania paradoxa* predation on *Aedes aegypti*.

*Phyllocrania paradoxa* readily preyed on *Ae. aegypti* mosquitoes. This mantis used sit-and-wait and active hunting modes to capture its preys (Videos 1 and 2). All individuals caught about five mosquitoes (50%) within 60 min and around ten mosquitoes in 210 min (Fig. 1). All the mosquitoes (10) were caught within 24 h. A similar capacity of preying was observed between the predators. Indeed, the risk of death of mosquitoes was similar with all *P. paradoxa* individuals (Cox model: χ^2^ = 1.601, df = 2, p = 0.4491; Supplementary Table S1).

#### Dynamics of mantis predation on marked *Aedes albopictus* mosquitoes

*Phyllocrania paradoxa* preyed on both marked and unmarked male *Ae. albopictus.* These mosquito treatments showed similar vulnerability to mantis predation (Cox model: χ^2^ = 1.3071 df = 2, p = 0.5202, Supplementary Table S2). Fifty per cent of mosquitoes were caught within 150 min (Supplementary Fig. S1) and all were caught within 24 h.

#### Effect of marking on male *Aedes* vulnerability to mantis predation

*Hymenopus coronatus* 1, *P. paradoxa* 1 and *P. paradaoxa* 2 preyed similarly on unmarked and marked male *Ae. aegypti* (GLMM, Mantis: χ^2^ = 3.1155, df = 2, p = 0.2106, Mosquito: χ^2^ = 0.1308, df = 1, p = 0.7176). Overall, less than 60% of each mosquito treatment were caught within one hour (Supplementary Fig. S2).

#### Relative vulnerabilities of *Aedes aegypti, Aedes albopictus* and *Anopheles arabiensis* species to mantis predation

Within each sex, *Ae. aegypti* and *Ae. albopictus* were similarly vulnerable to *P. paradoxa* predation (p > 0.05, Supplementary Table S3; Supplementary Fig. S3a,b). For female mosquitoes, no difference was observed between mantis individuals in the number of mosquitoes caught (p > 0.05, Supplementary Table S3). However, for males, *P. paradoxa* 3 caught more mosquitoes than *P. paradoxa* 2 (Supplementary Fig. S3a; Odds ratio ± se = 0.274 ± 0.116; z. ratio = − 3.060; p = 0.0022).

Females of *Ae. aegypti* and *An. arabiensis* were similarly predated by both *B. mendica* and *H. coronatus* 2 (χ^2^ = 0.0308, df = 1, p = 0.8607; Supplementary Table S3)*.* However, *B. mendica* caught more mosquitoes than *H. coronatus* 2 overall (Odds ratio ± se = 3.67 ± 1.36; z. ratio = 3.506, p = 0.0005; Supplementary Fig. S4a).

Mosquito species was found to affect the predation rate in the comparison of *Ae. albopictus* and *An. arabiensis (*χ^2^ = 5.2805, df = 1, p = 0.02157; Supplementary Fig. S4b). Female *Ae. albopictus* were significantly more caught than female *An. arabiensis* (Odds ratio ± se = 2.06 ± 0.633, z. ratio = 2.362; p = 0.0182). No difference was observed between mantis individuals *(*χ^2^ = 2.8374, df = 2, p = 0.24203; Supplementary Table S3).

#### Relative vulnerabilities of male and female mosquitoes to mantis predation

*Blepharopsis mendica* and *H. coronatus* 2 and *P. paradoxa* 3 similarly predated male and female mosquitoes within *Ae. albopictus* species (Supplementary Fig. S5a; GLMM, Mantis: χ^2^ = 3.5950, df = 2, p = 0.1657, Mosquito sex: χ^2^ = 1.3470, df = 1, p = 0.2458). Within *Ae. aegypti* species*,* these mantises caught more males than females (Odds ratio ± se = 2.71 ± 0.776, z. ratio = 3.493, p = 0.0005; Supplementary Fig. S5b). Overall, *B. mendica* and *H. coronatus* 2 showed similar predation rates (Odds ratio ± se = 1.12 ± 0.37; z. ratio = 0.334, p = 0.9404) and both were statistically higher than that of *P. paradoxa* 3 (*B. mendica /P. paradoxa* 3: Odds ratio ± se = 5.28 ± 1.91; z. ratio = 4.609, p < 0.001; *H. coronatus* 2 */P. paradoxa* 3: Odds ratio ± se = 4.73 ± 1.70; z. ratio = 4.331, p < 0.001). Within *An. arabiensis* (Supplementary Fig. S5c), predation rate was not affected by mosquito sex (χ^2^ = 1.6551, df = 1, p = 0.1983) and only *B. mendica* significantly caught more mosquitoes than *P. paradoxa* 3 (Odds ratio ± se = 3.27 ± 1.35; z. ratio = 2.863, p = 0.0117; Supplementary Table S4).

#### Effect of chilling on male *Aedes albopictus* vulnerability to mantis predation

All the mantises preyed on chilled and non-chilled *Ae. albopictus* males with up to 60% mosquitoes caught per treatment within 60 min (Supplementary Fig. S6). No significant difference was observed between mosquito treatments (χ^2^ = 2.1668, df = 1, p = 0.1410). Only *B. mendica* showed a slightly higher predation rate than *H. coronatus* 1 (Odds ratio ± se = 4.49 ± 2.48; z. ratio = 2.478, p = 0.0503; Supplementary Table S5).

#### Effect of irradiation on male *Aedes* vulnerability to mantis predation

**Figure 2:**
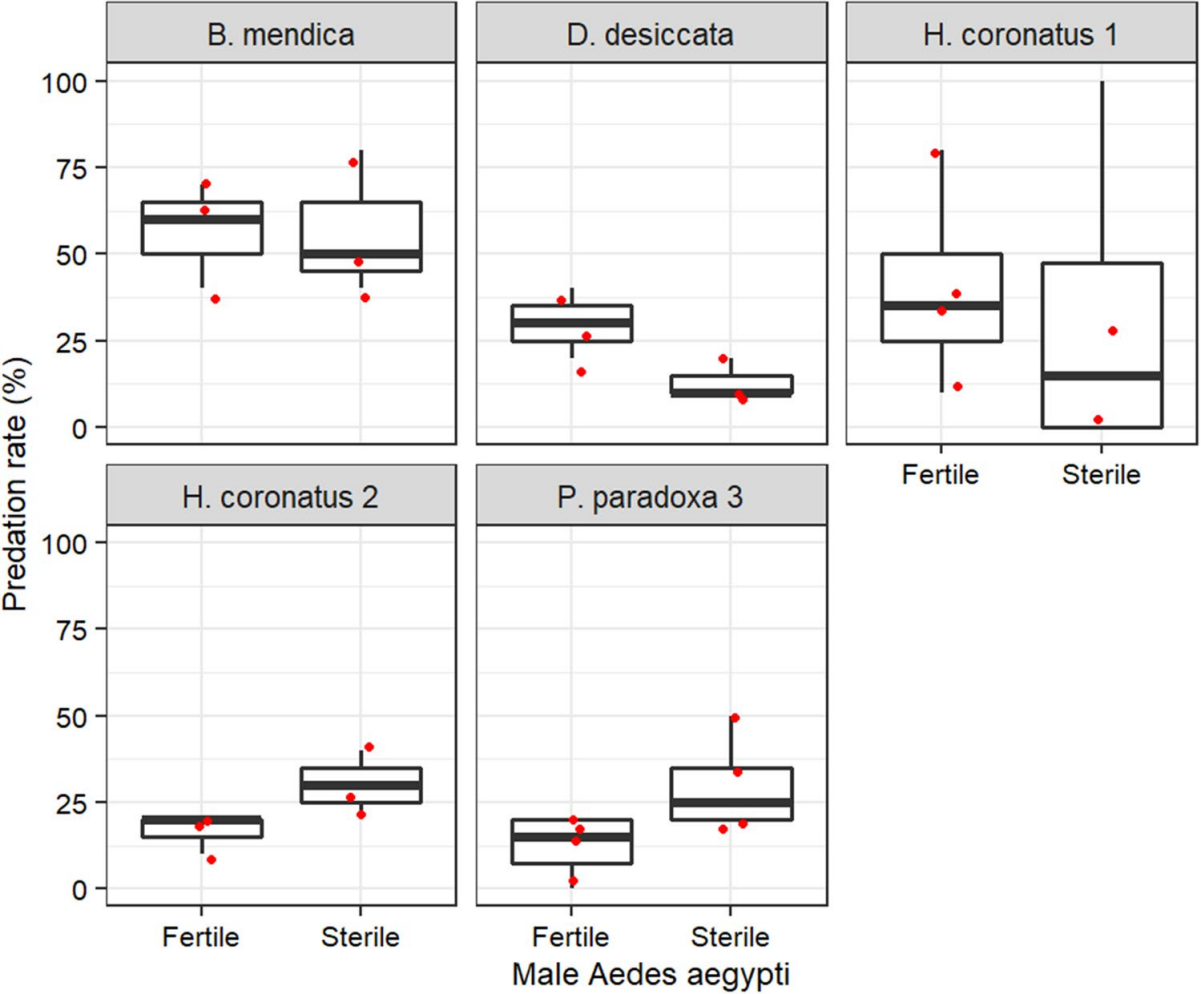
Vulnerability of fertile versus sterile male *Aedes aegypti* (irradiated at 70 Gy) to mantis predation.

The dosimetry confirmed that all doses received lay within a 5% error range. All the mantises preyed on irradiated and unirradiated males of both *Aedes* species. For *Ae. aegypti,* except *B. mendica,* all mantises mostly caught less than 50% mosquitoes per treatment within 60 min (Fig. 2). Significant effect was observed on factor mantis and on its interaction with mosquito treatment but not on the mosquito treatment (Supplementary Table S6). *Blepharopsis mendica* caught more mosquitoes, fertile or sterile ones, than the other predators (Supplementary Table S7). However, *P. paradoxa* 3 caught marginally more sterile males than fertile ones (GLMM, Emmeans, p = 0.0499; Supplementary Table S7) whereas *D. desiccata* caught more fertile males (GLMM, Emmeans, p = 0.0355; Supplementary Table S7) and no difference was observed with the other mantises (GLMM, Emmeans, p > 0.05; Supplementary Table S7).

**Figure 3:**
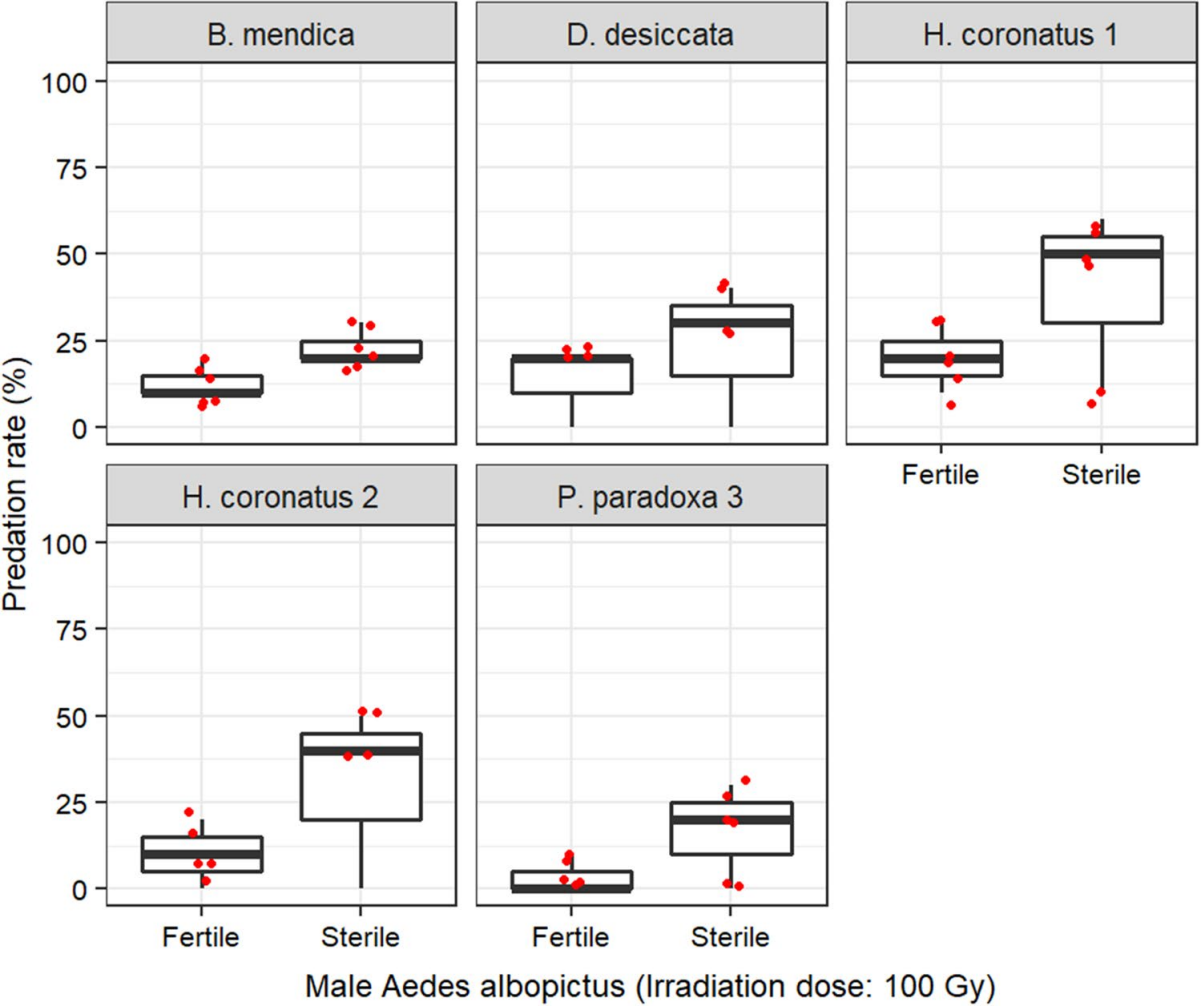
Vulnerability of fertile versus sterile male *Aedes albopictus* (irradiated at 100 Gy) to mantis predation.

For *Ae. albopictus*, predation rate was less than 50%. When males were irradiated with 65 Gy, a significant effect was only observed in the interaction between the mantis factor and the mosquito treatment (Supplementary Table S8). *Phyllocraniaparadoxa* marginally caught more fertile than sterile males (Odds ratio ± se = 4.215 ± 2.44, z. ratio = 2.480, p = 0.0131) and *H. coronatus* 1 similarly caught fertile and sterile males (Odds ratio ± se = 0.568 ± 0.273, z. ratio = − 1.179, p = 0.2386; Supplementary Fig. S7). In contrast, when males were irradiated with 100 Gy, no significant difference was observed between predators (χ^2^ = 5.8281, df = 4, p = 0.2124) and all of them caught more sterile males than fertile ones (Odds ratio ± se = 0.392 ± 0.124; z. ratio = − 2.967, p = 0.0030; Fig. 3).

### Gecko predation propensity on *Aedes* and *Anopheles* mosquitoes

#### Predatorial behavior of *Phelsuma standingi* on *Aedes* and *Anopheles* mosquitoes

**Figure 4:**
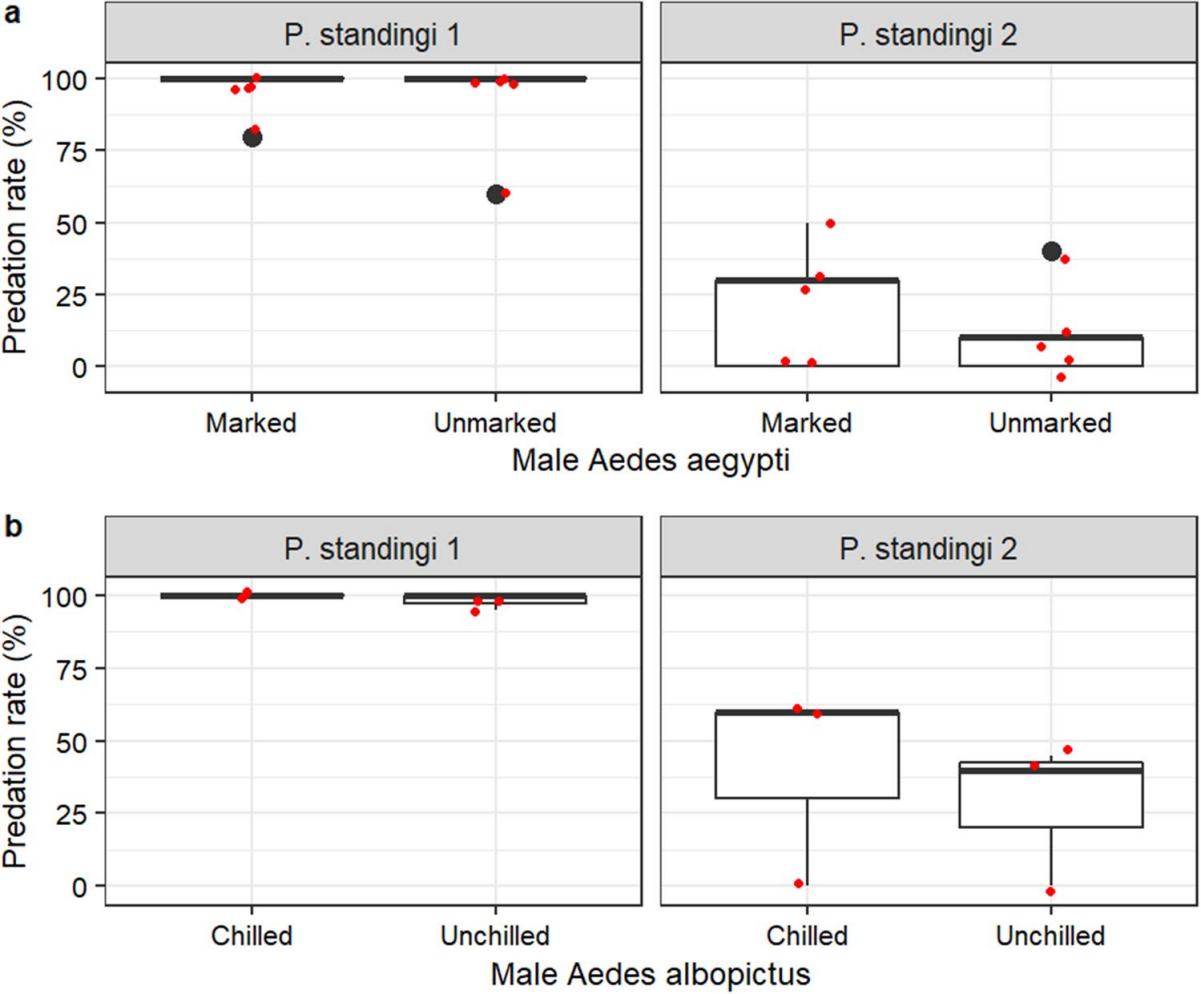
Effect of mosquito marking or chilling on their vulnerability to *Phelsuma standingi* predation. (**a**) marked *versus* unmarked male Aedes aegypti. (**b**) Chilled *versus* non chilled male *Aedes albopictus*.

**Figure 5:**
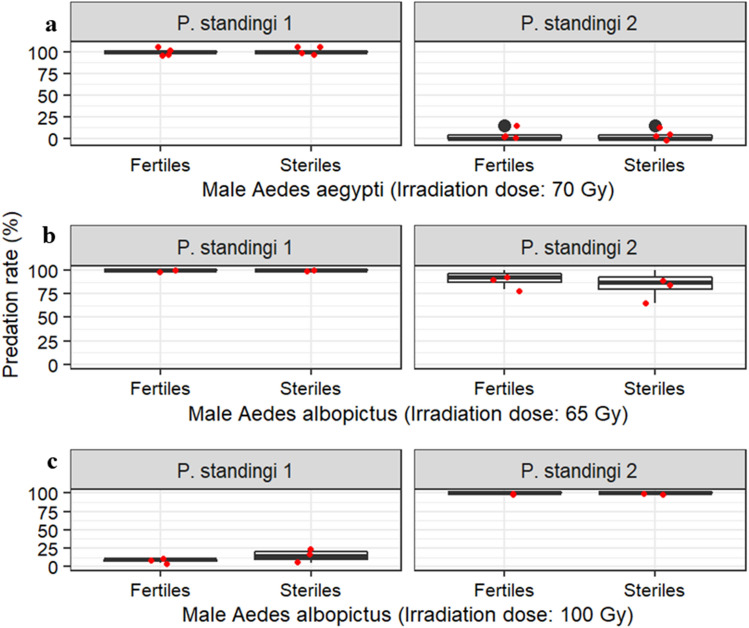
Vulnerability of fertile versus sterile male *Aedes* males to *Phelsuma standingi* predation. (**a**) *Aedes aegypti* with irradiation dose of 70 Gy; (**b**) *Aedes albopictus* with irradiation dose of 65 Gy; (c: *Aedes albopictus* with irradiation dose of 100 Gy.

**Figure 6:**
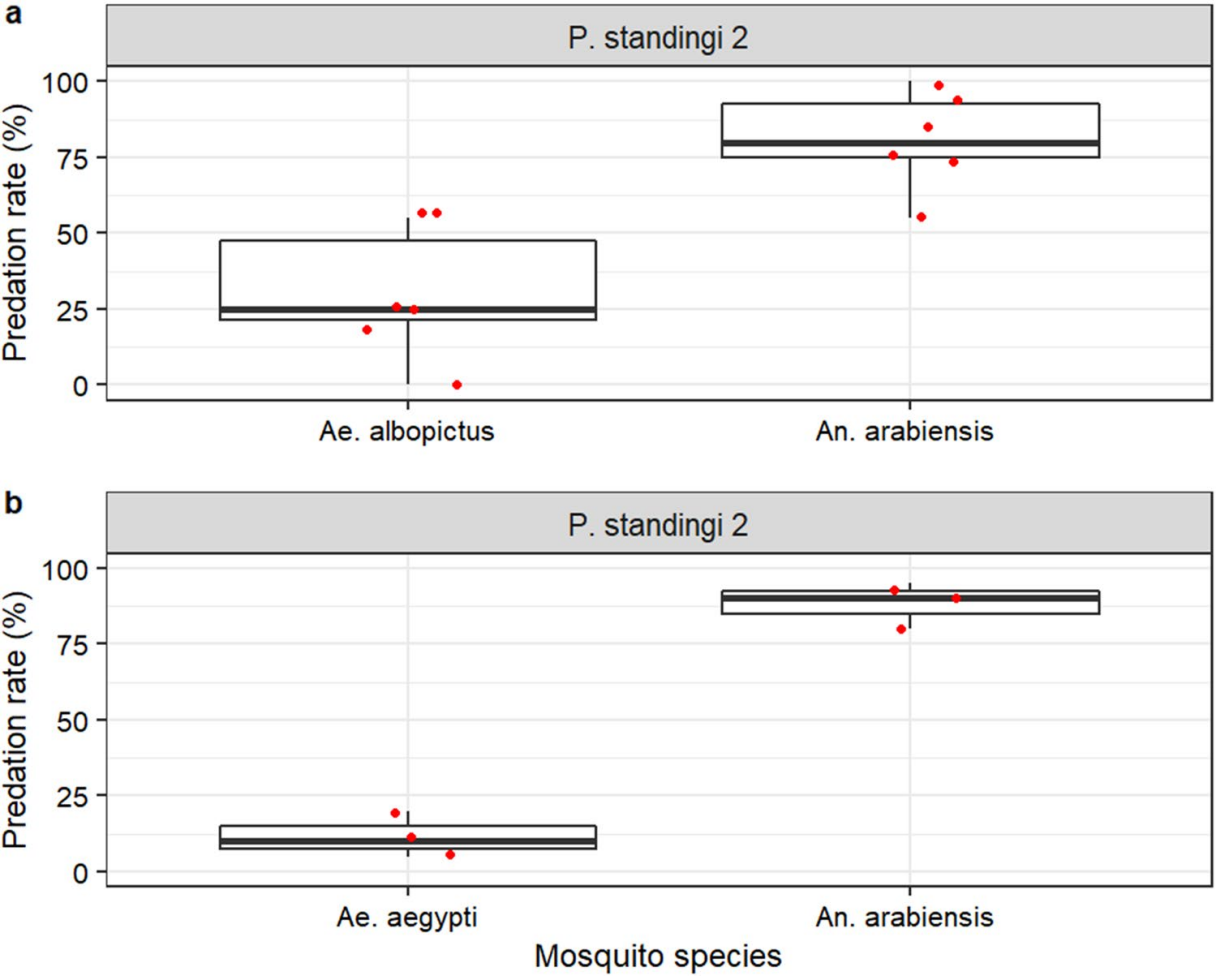
*Phelsuma standingi* predation preference for *Aedes* or *Anopheles* mosquitoes. (**a**) *Ae. albopictus versus Anopheles arabiensis;* (**b**) *Ae. aegypti versus Anopheles arabiensis*.

*Phelsuma standingi* showed high predation rate on mosquitoes (Video 3), up to 100% (40 mosquitoes) within an hour. Similar vulnerability to gecko predation was observed for the following compared mosquito treatments: marked *vs* unmarked male *Ae. aegypti* (GLMM, χ^2^ = 2.8607, df = 1, p = 0.0908; Fig. 4a), chilled *vs* non chilled male *Ae. albopictus* (χ^2^ = 1.0886, df = 1, p = 0.2968; Fig. 4b), male *vs* female *Ae. aegypti* (χ^2^ = 3.1709, df = 1, p = 0.0750; Supplementary Fig. S8a), male *vs* female *An. arabiensis* (χ^2^ = 0.9445, df = 1, p = 0.3311; Supplementary Fig. S8b), fertile *vs* sterile male *Ae. aegypti* (χ^2^ = 0.0904, df = 1, p = 0.7637; Fig. 5a), fertile *vs* sterile male *Ae. albopictus* irradiated at 65 Gy (χ^2^ = 1.4262df = 1, p = 0.2324; Fig. 5b) or at 100 Gy (χ^2^ = 1.0763, df = 1, p = 0.2995; Fig. 5c). However, *P. standingi* showed a marked preference for *An. arabiensis* as compared to *Ae. aegypti* (GLMM, Emmeans, Odds ratio ± se = 62.8 ± 38.6; z. ratio = 6.743, p < 0.0001; Fig. 6a), and to *Ae. albopictus* (Odds ratio ± se = 0.0643 ± 0.0238; z. ratio = 4.498, p < 0.0001; Fig. 6b).

Considering the total number of mosquitoes caught, *P. standingi* 1 was significantly more effective than *P. standingi 2* in the tests *“*marked *vs* unmarked male *Ae. aegypti* (Odds ratio ± se = 641 ± 833; z. ratio = 4.977, p < 0.001; Fig. 4a), “chilled *vs* non-chilled male *Ae. albopictus* (Odds ratio ± se = 592 ± 646; z. ratio = 5.844, p < 0.001: Fig. 4b), fertile *vs* sterile male *Ae. aegypti* (Odds ratio ± se = 1343 ± 962; z. ratio = 10.058, p < 0.001; Fig. 5a), “fertile *vs* sterile male *Ae. albopictus* irradiated at 65 Gy” (Odds ratio ± se = 11.400 ± 0.068; z. ratio = 406.955, p < 0.001; Fig. 5b). In contrast, *P. standingi* 2 showed a higher predation rate than *P. standingi* 1 in the test “fertile *vs* sterile male *Ae. albopictus* irradiated at 100 Gy” (Odds ratio ± se = 447 ± 343; z. ratio = 7.948, p < 0.001; Fig. 5c).

#### Relative vulnerabilities of different mosquito species to *Phelsuma laticauda* predation

**Figure 7:**
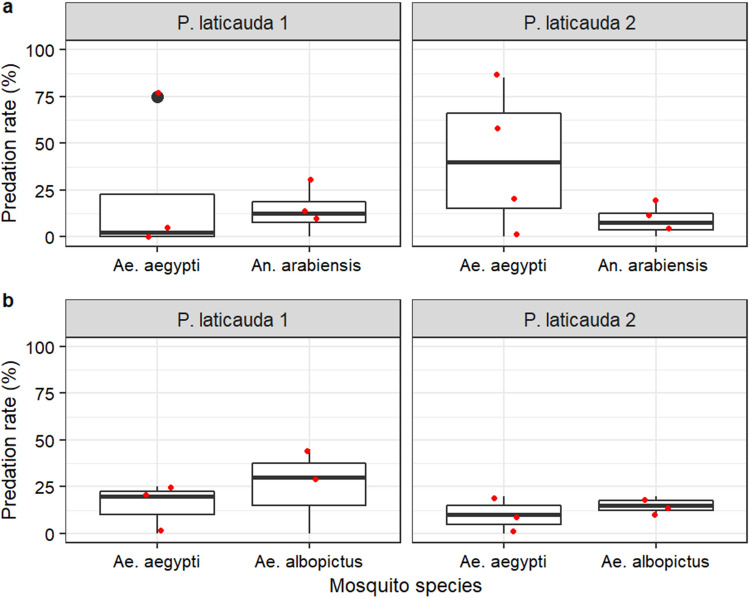
Effect of mosquito species on *Phelsuma laticauda* predation. (**a**) *Aedes aegypti* versus *Anopheles arabiensis;* (**b**) *Aedes aegypti versus Aedes albopictus*.

When *Ae. aegypti* and *An. arabiensis* were offered to *P. laticauda* together, predation rates were less than 40% for *An. arabiensis* and ranged between 0 and 90% for *Ae. aegypti* (Fig. 7a). No difference was observed in the total number of mosquitoes caught between *P. laticauda* 1 and *P. laticauda 2* (GLMM: χ^2^ = 3.288, df = 1, p = 0.070). However*, P. laticauda* 2 caught more *Ae. aegypti* than *An. arabiensis* (GLMM, Emmeans, Odds ratio ± se = 8.15 ± 3.803; z. ratio = 4.498, p < 0.001) while no difference was observed with *P. laticauda* 1 (Odds ratio ± se = 1.60 ± 0.697; z. ratio = 1.078, p = 0.2811).

When *Ae. aegypti* and *Ae. albopictus* were offered together, predation rates were less than 50% for gecko individuals (Fig. 7b). No difference was observed in the predation rate between *P. laticauda* 1 and *P. laticauda 2* (χ^2^ = 2.598, df = 1, p = 0.1070)*,* and between mosquito species (χ^2^ = 2.598, df = 1, p = 0.1070).

## Discussion

**Figure 8:**
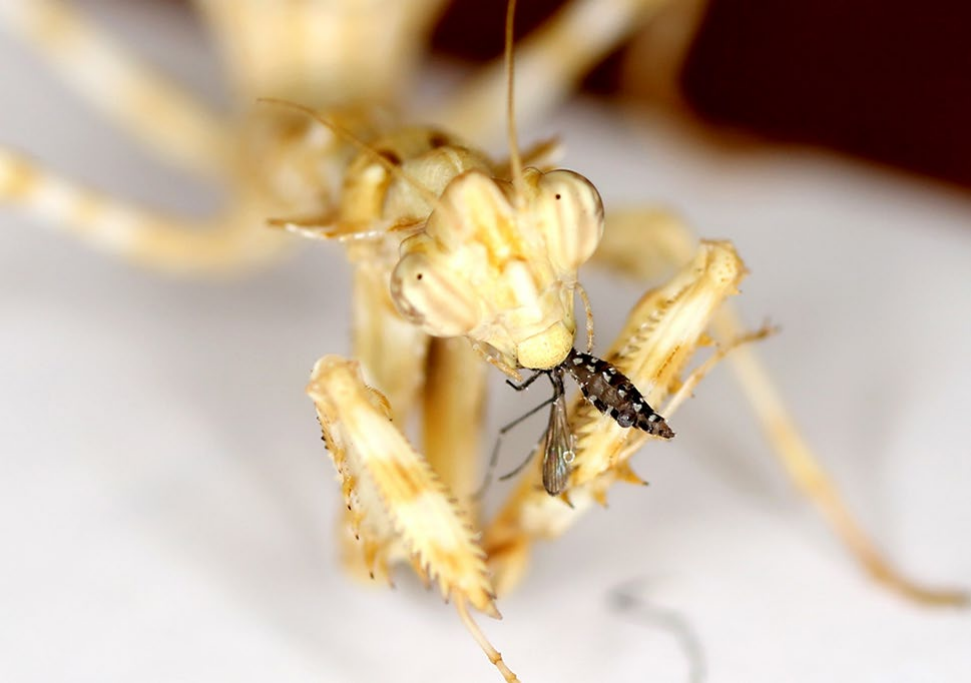
*Blepharopsis mendica* eating a female *Aedes* mosquito. (Photo credit: Wallner T.).

The results of the study showed that the predation propensity and prey preferences of the tested predators are diverse, likely mirroring their naturally encountered prey according to their native environment and behavioural activities. The mantises effectively preyed on *Aedes* and *Anopheles* mosquito species, indicating that mosquitoes are part of these mantis species diet (Fig. 8).

All the mantises were able to eat more than ten mosquitoes within an hour. Neither mosquito species and sex, nor marking and chilling affected the mosquito vulnerability to mantis predation under our study conditions. However, the effect of irradiation depended on the dose. Indeed, irradiation with 100 Gy led to more vulnerable mosquitoes as compared to unirradiated mosquitoes, whereas the doses of 65 Gy or 70 Gy mainly had no impact or even led to less vulnerable mosquitoes. It is known that prey speed influences susceptibility to predation. Since mantises are mainly sit-and-wait predators^53^, chances for a mosquito to escape from predation may rely on its flight speed. Adverse effects of high irradiation doses on male mosquito quality including competitiveness and flight ability have previously been reported^44,45,54,55^. The vulnerability of males irradiated with 100 Gy could therefore be related to reducing of their flight ability. This result indicates that high irradiation doses can expose sterile males to increased predation and then compromise the success of the SIT hence the necessity to adjust the optimal irradiation dose. However, 100 Gy is a very high dose for this species and nearly double that is needed for full sterility. The 100 Gy irradiation treatment served as verification that highly negatively affected mosquitoes, with debilitating treatment consequences are indeed more prone to predation, meaning that the actual dose applied in this species for near full sterility does not increase risk of predation- at least in artificial settings.

Mantis predation may be considered as a potential tool for quality control for laboratory-produced mosquitoes. Furthermore, our study showed that mantis predation propensity on mosquitoes is driven by complex factors that need to be elucidated. Indeed, predation rate varied between mantis species, individuals from the same mantis species, and over days of predation. In some tests, the mantises presented similar feeding rates while in others, significant differences were observed. The development stage (age) and especially molting periods and the sex of the predators may play an essential role in this variation as they were of different ages and may have different food requirements at a given time. Indeed, low predation rates (0% or nearly) were mainly recorded during molting periods. Overall and regardless of the treatment, *B. mendica* showed more predatory capacities as compared to the other mantis species. The use of this species could be further investigated as a quality control tool for sterile male mosquitoes.

It is has been reported that most predatory mantises exhibit a sexual cannibalism with females selectively eating the smaller males^56,57^. Although our study did not aim to investigate this question, cannibalism was noticed with *P. paradoxa* species with the loss of *P. paradoxa* 1 when the three individuals were maintained together in the same cage.

In general, the food web of geckos and mantises includes various species of insects including mosquitoes. Many studies carried out on house gecko feeding behavior, *Hemidactylus frenatus* and *H. platyurus,* showed that they feed mostly on Diptera with a preference for *Lepidoptera* and *Culicidae*^18,58,59^. However, literature research about the day geckos *P. standingi* and *P. laticauda* predation on mosquitoes was not found. Here, our study clearly showed that these geckos feed on *Ae. aegypti*, *Ae. albopictus* and *An. arabiensis* adult mosquitoes. Moreover, mosquito chilling, marking and irradiation procedures did not impact the laboratory-reared mosquito vulnerability to the standing’s day gecko predation. *Phelsuma standingi* demonstrated a relatively high predation rate, since it caught all or almost all of the mosquitoes provided (40 mosquitoes), like the Australian gecko *Gehydra dubia* and the exotic Asian house gecko *H. frenatus* which were found to consume 63–109 *Aedes aegypti* within 24 h in a similar study^16^. However, in contrast to these studies, no difference was observed between mosquito sexes in their vulnerability to *P. standingi* predation*.* The geckos used sit-and-wait for prey and active hunting predation modes to capture the mosquitoes and this may partly explain the high predation rates observed. However, like with mantises, we found that the predation rate significantly varied over time and between the two gecko individuals: at the beginning of the study, *P. standingi* 1 caught almost all of the mosquitoes provided (40 mosquitoes) while *P. standingi* 2 caught only a few, regardless of the treatment but the inverse was later observed in some tests. Critical factors such as the development stage (age) and the molting period and the sex of the gecko may also play an important role in the predation propensity. For example, Dor et al*.*^60^ found that females of the lizard, *Norops serranoi*, exhibited higher predation pressure and were more efficient at capturing the Mexican fruit fly, *Anastrepha ludens*, than males. Furthermore, our study showed that *P. standingi* predation propensity was affected by mosquito prey species. Indeed, when only one mosquito species was offered to this gecko, similar predation rates were observed between species. But when *Anopheles* and *Aedes* were offered together, the standing’s day gecko clearly showed preference for *An. arabiensis* than *Ae. aegypti* (80–95% *vs* 5–20%), and *Ae. albopictus* (55–100% *vs* 0–55%). However, the preference of *P. laticauda* for *Aedes* or *Anopheles* mosquito species is not clear since one individual showed similar predation rate on *Ae. aegypti* and *An. arabiensis* while another individual presented a preference for *Ae. aegypti*. This gecko was found to catch *Ae. albopictus* and *Ae. aegypti* similarly*.* The determinants of the gecko preference for a given mosquito species remains to be investigated. Like any generalist predator, gecko prey selection may be influenced by a wide range of factors including prey size, softness, taste, nutritive value, energy requirement to capture the prey and adaptive traits^61,62^. Indeed, gecko’s preference between mosquito species relying on their adaptive traits was previously reported by Canyon and Hii^16^ who observed that *G. dubia* preferred eating photophilic species, *Ae. vigilax, An. annulipes, Coquillettidia xanthogaster, Culex annulirostris, Cx. sitiens* (78–100%) compared to non-photophilic species *Ae. aegypti, Ae. notoscriptus, Ae.vittiger* and *Cx. quinquefasciatus* (33–53%).

Overall, our study has increased the cohort of known adult mosquito predators, which were not considered in the review of Collins et al.^63^. In addition, it has provided basic information about predation and factors that may influence predation risk of laboratory-produced sterile males. However, our results are not sufficient to predict their relationships with wild predators in the field since the study was carried out under laboratory conditions using laboratory-maintained preys and predators which are not accustomed to prey-predator interactions. Indeed, in our settings, mosquitoes had less chance to escape from predation since they were caged (30 × 30 × 30 cm for geckos and 15 × 15 × 15 cm for mantises) and could not undertake long-distance flights. In addition, the assessed predators are known to be polyphagous and their predation propensity on mosquitoes in field conditions may be influenced by other sources of food (nectar and other preys) and competitors and environmental factors. Therefore, investigations need to be enhanced by comparing, at least under semi-field conditions, the vulnerability of laboratory-produced mosquitoes and their field-collected counterparts to wild predators.

## Material and methods

### Biological material

#### Mosquito species used as preys

*Aedes aegypti*, *Ae. albopictus* and *An. arabiensis* mosquitoes were used as preys in this study. The strains used were maintained at the Insect Pest Control Laboratory (IPCL) of the joint FAO/IAEA Centre of Nuclear Techniques in Food and Agriculture, Seibersdorf, Austria under laboratory settings following the rearing guidelines developed at the IPCL (FAO/IAEA, 2017a, FAO/IAEA, 2017b). The *Ae. aegypti* strain originated from Juazeiro, Brazil and was provided to the IPCL in 2012 by Biofabrica Moscamed, a collaborative centre of the IAEA on the development of the SIT against mosquitoes. The *Ae. albopictus* colony originated from Rimini in Italy and was provided to the IPCL in 2018 by Centro Agricoltura Ambiente, a collaborative centre of the IAEA on the development of the Sterile Insect Technique against mosquitoes. The *An. arabiensis* strain originated from Dongola in the Northern State of Sudan and was provided to the IPCL by the Tropical Medicine Research Institute, Khartoum, in 2005.

#### Assessed predators

The predators under investigation in this study included four mantis species, *Phyllocrania paradoxa, Hymenopus coronatus, Blepharopsis mendica, Deroplatys desiccata,* and two gecko species, the standing’s day gecko, *Phelsuma standingi,* and *P. laticauda*. All mantises were used at their larval stage.

The characteristics of the predators are presented in Table 1. Preys and predators used in this study were kept in the same laboratory, where predation experiments were carried out. The environmental conditions in the laboratory were 26 ± 2 °C, 60 ± 10%RH, 14 h:10 h light: dark, including 1 h dawn and 1 h dusk. During the whole study period, the predators were fed only with adult mosquitoes. Predation tests were carried out on adult mosquitoes only. Predators were starved 3 days before every test. Tests performed on different days on a same predator individual were considered as replicates. Individuals from the same predator species were taken as different since they might be of different age or sex.

**Table 1:** Description of the predators.

Predators	Predator Species	Habitat, temperature (^o^C), relative humidity (RH), size (cm)	Distribution	Quantity	Provider
Mantis	*Phyllocrania paradoxa*	Terratium, 20–30 °C, 50–60% RH, up to 5 cm	Africa, Madagascar	3	Megazoo, Vienna, Austria
	*Hymenopus coronatus*	Terratium, 30–35 °C, 70–100% RH, up to 7 cm	India, Malaysia, Thailand	2	Megazoo, Vienna, Austria
	*Blepharopsis mendica*	Terratium, 30–40 °C, 40–60% RH, up to 7 cm	Canary Islands, Middle East, North Africa	1	Megazoo, Vienna, Austria
	*Deroplatys desiccata*	Terratium, 25–30 °C, 60–80% RH, up to 8 cm	Borneo, Java, Malaysia, Sumatra	1	Megazoo, Vienna, Austria
Gecko	*Phelsuma standingi*	Savana, 25–30 oC, 60–90% RH, up to 28 cm	Madagascar	2	Megazoo, Vienna, Austria
	*Phelsuma laticauda*	Savana, 20–28 °C, 65–75% RH, 15–22 cm	Madagascar, Comoros	2	Megazoo, Vienna, Austria

### Investigation on mantis predation propensity on *Aedes* and *Anopheles* mosquitoes

All the tests were performed using BugDorm cages (15 × 15 × 15 cm), (MegaView Science Co. Ltd., Taichung 40762, Taiwan). Every cage contained a single predator.

Preliminary tests were carried out to determine whether mantises prey on both marked, and unmarked mosquitoes, and determine the predation rate over time. Thereafter, predation preference between different mosquito species and characteristics was evaluated.

#### Assessment of the dynamics of mantis predation on *Aedes* mosquitoes

Ten females *Ae. aegypti* were offered to every *P. paradoxa* in individual BugDorm cages. The number of remaining mosquitoes was recorded every 30 min for 210 min and after 24 h. Three individuals of *P. paradoxa*, named *P. paradoxa* 1, *P. paradoxa* 2, and *P. paradoxa* 3, were involved in this study. Two replicates were performed per individual.

#### Assessment of the dynamic of mantis predation on marked *Aedes* mosquitoes

Ten marked male *Ae. albopictus* were offered only to *P. paradoxa* 1. Both *P. paradoxa* 2 and *P. paradoxa* 3 were kept as controls with 10 unmarked male *Ae. albopictus* each. The number of remaining mosquitoes was recorded every 30 min for 210 min and after 24 h. Three replicates were performed. To mark, the mosquitoes were chilled for 5 min at 4 °C in a fridge and marked with pink fluorescent pigment (RADGLO JST, Radiant NV, Houthalen, Belgium or DayGlo Color Corp., Cleveland, USA) at the dose of 5 mg/1000 mosquitoes^64^. The control mosquitoes were chilled simultaneously for the same duration but not marked.

In the following experiments on mantis predation, we assessed their predation preference between different treatments of mosquitoes. Mosquitoes were offered to the mantises for one hour. Then, the mantises were removed from the cages, and the remaining mosquitoes were killed by freezing, identified and counted by treatment under a stereomicroscope.

#### Relative vulnerabilities of *Aedes aegypti, Aedes albopictus* and *Anopheles arabiensis* species to mantis predation

Relative vulnerability of *Aedes* species to mantis predation was evaluated by sex. Ten *Ae. albopictus* and 10 *Ae. aegypti* of the same sex were provided at once to every individual mantis. For female mosquitoes, three replicates were performed with *P. paradoxa* 1, *P. paradoxa* 2, and *P. paradoxa* 3*.* For male mosquitoes, three replicates were performed with *P. paradoxa* 2, and *P. paradoxa* 3.

Relative vulnerability of *Aedes* and *Anopheles* species to mantis predation were assessed; first, between *Ae. albopictus* and *An. arabiensis*, and second, between *Ae. aegypti* and *An. arabiensis*. For each comparison*,* 10 females *Aedes* (*Ae. albopictus* or *Ae. aegypti*) and 10 female *An. arabiensis* were offered at once to every individual mantis in a cage. Four replicates were performed with *P. paradoxa* 3, *H. coronatus* 2 and *B. mendica* in both part of the study.

#### Effect of marking on male *Aedes* vulnerability to mantis predation

Ten marked and 10 unmarked males *Ae. aegypti* were offered at once to every individual mantis. Four replicates were performed with *P. paradoxa* 2, *P. paradoxa* 3 and *H. coronatus* 1*.* Mosquitoes were marked as described above.

#### Effect of chilling on male *Aedes* vulnerability to mantis predation

Tests were carried out on male *Ae. albopictus* chilled at 4 °C for 2 h in a fridge. The chilled mosquitoes were marked with pink fluorescent pigments^64^ and kept in the laboratory for one hour before the test to allow recovery. The non-chilled mosquitoes were not marked. Ten chilled and 10 non-chilled males were offered at once to every individual mantis in a cage. Three replicates were performed with *P. paradoxa* 3*, H. coronatus* 1, *H. coronatus* 2, *B. mendica,* and *D. desiccata.*

#### Effect of irradiation on male *Aedes* vulnerability to mantis predation

A self-contained ^60^Co Gammacell 220 (Nordion Ltd, Kanata, Ontario, Canada) irradiator was used for mosquito irradiation. The dosimetry system used to verify the dose received by the batches was based on GafChromic HD-V2 (Ashland Advanced Materials, Bridgewater NJ, USA). Three films of HD film were packed in individual envelopes and placed directly below the adult samples. The temperature near the sample and films was measured before and after radiation exposure. Films were read with an optical density reader after 24 h of development.

Adult mosquitoes were chilled for 5 min in a refrigerator and then transferred to a plastic box for irradiation. Subsequently, the mosquitoes were kept in the laboratory for, at least, one hour before the tests. Control mosquitoes were taken from the batch of chilled mosquitoes. Tests were performed on both *Ae. aegypti,* and *Ae. albopictus.* For each mosquito species, 10 irradiated and 10 unirradiated males were offered at once to every individual mantis in separate cages. Irradiated and unirradiated males were alternatively marked in the different repetitions using pink, fluorescent dust pigment described previously to ease identification. For *Ae. aegypti,* four replicates were performed with *P. paradoxa 3,* and *H. coronatus 1,* and three replicates with *B. mendica, H. coronatus 2,* and *D. desiccata.* For *Ae. albopictus,* tests were performed four times with *P. paradoxa* 3and *H. coronatus* 1. Mosquitoes were irradiated at the adult stage with 70 Gy for *Ae. aegypti* and 65 Gy for *Ae. albopictus.* The doses induce a level of sterility exceeding 99% in these species (unpublished data from another study). To evaluate better the effect of irradiation on mosquito vulnerability to mantis predation, further tests were performed using male *Ae. albopictus* irradiated at 100 Gy, a very high dose expected to induce debilitating biological effects, against unirradiated males*.* Three replicates were performed with *P. paradoxa* 3*, H. coronatus* 1, *H. coronatus* 2, *B. mendica,* and *D. desiccata.*

#### Relative vulnerabilities of male and female mosquitoes to mantis predation

Tests were performed on *Ae. albopictus, Ae. aegypti* and *An. arabiensis* species*.* For every mosquito species, 10 males and 10 females were offered at once to every individual mantis in separate cages. For *Ae. aegypti*, five replicates were performed with *P. paradoxa* 3, *H. coronatus* 2 and *B. mendica.* For *Ae. albopictus* and for *An. arabiensis,* three replicates were performed with *P. paradoxa* 3, *H. coronatus* 2 and *B. mendica.*

### Investigation on the predation propensity of the geckos, *Phelsuma standingi* and *Phelsuma laticauda, *on *Aedes* and *Anopheles* mosquitoes

#### Relative vulnerabilities of different mosquito species and treatments to *Phelsuma standingi* predation

Predation propensity of the standing’s day gecko, *Phelsuma standingi,* on different mosquito species and treatments was evaluated. Two individuals of *P. standingi,* hereafter named *P. standingi* 1, and *P. standingi* 2, were involved in this study. The gecko’s predation propensity was assessed for: (1) marked *versus* unmarked male *Ae. aegypti*: five replicates were done for both geckos; (2) chilled (at 4 °C for 2 h) *versus* non-chilled male *Ae. albopictus*: three replicates were done for both geckos; (3) fertile *versus* sterile male *Ae. aegypti,* irradiated at 70 Gy): four replicates were done with both geckos; (4) fertile *versus* sterile male *Ae. albopictus*, irradiated at 65 Gy (4 replicates) or 100 Gy (3 replicates) with both geckos; (5) male *versus* female *Ae. aegypti*: only *P. standingi* 2 was evaluated*,* and three replicated were performed; (6) female *Ae. albopictus versus* female *An. arabiensis*: only *P. standingi* 2 was evaluated, and six replicates were performed; (7) female *Ae. aegypti versus* female *An. arabiensis*: only *P. standingi* 2 was evaluated, and three replicates were performed; (8) female *versus* male *An. arabiensis*: only *P. standingi* 2 was evaluated, and three replicates were performed. All experiments were conducted using BugDorm cages (30 × 30 × 30 cm). Geckos were kept in separated cages. In all experiments, 20 mosquitoes from each treatment (except in the test 1 where 10 mosquitoes per treatment were used) were pooled and offered to each gecko in a cage for one hour. Subsequently, the geckos were removed from the cages, and the remaining mosquitoes were frozen, identified and counted by treatment. The procedures for mosquito chilling and marking mentioned in the experiments with mantises, apply in the ones with the geckos.

#### Relative vulnerabilities of different mosquito characteristics to *Phelsuma laticauda* predation

Predation propensity of the gold dust day gecko, *P. laticauda,* on different mosquito species was assessed for: (1) female *Ae. aegypti versus* female *An. arabiensis* and (2) female *Ae. albopictus versus* female *Ae. aegypti*. The two individuals of *P. laticauda* named *P. laticauda* 1, and *P. laticauda* 2 were used in both experiments. For each gecko, four replicates were performed for experiment 1, and three replicates for experiment 2. In each experiment, 20 females from each mosquito species were pooled and offered to each gecko in a BugDorm cage (30 × 30 × 30 cm) for one hour. Afterward, the geckos were removed from the cages, and the remaining mosquitoes were frozen, identified and counted by species.

### Statistical analysis

Data were analyzed using R software (version 4.0 0.0). Mixed-effects cox regression models were used to analyze the risk of death of mosquitoes in the presence of predators. Predators were considered as fixed effect and the replicates as random effect. To compare the predation rate between predators and between different groups of mosquitoes, binomial generalised linear mixed models fit by maximum likelihood (Laplace Approximation) with the proportion of caught mosquitoes as response variable, predators (gecko or mantis), mosquito treatment as fixed effects and the replicates as random effect were used. When explanatory variables or their interaction presents a significant effect, pairwise comparisons were performed between the levels of the factors, using the “Emmeans” package and considering the best fit linear mixed model.

## Supplementary Information

Supplementary Information 1.

Supplementary Legends.

Supplementary Video 1.

Supplementary Video 2.

Supplementary Video 3.

## Data Availability

The datasets generated during the current study are available from the corresponding author on reasonable request.
