# Supplementary material for: Adult mosquito predation and potential impact on the sterile insect technique
Source: Sci Rep. 2022 Feb 15;12:2561. doi: 10.1038/s41598-022-06565-1 (PMC8847352; doi:10.1038/s41598-022-06565-1)
Supplement: Supplementary file 1 — Supplementary Information 1. [file 41598_2022_6565_MOESM1_ESM.pdf]

Adult mosquito predation and potential impact on the sterile insect technique

Nanwintoum Séverin Bimbilé Somda[a], Hamidou Maïga, Wadaka Mamai[a], Thierno Bakhoum, Thomas Wallner, Serge Bèwadéyir Poda, Hanano Yamada, Jérémy Bouyer

Supplementary Information

Supporting Figures

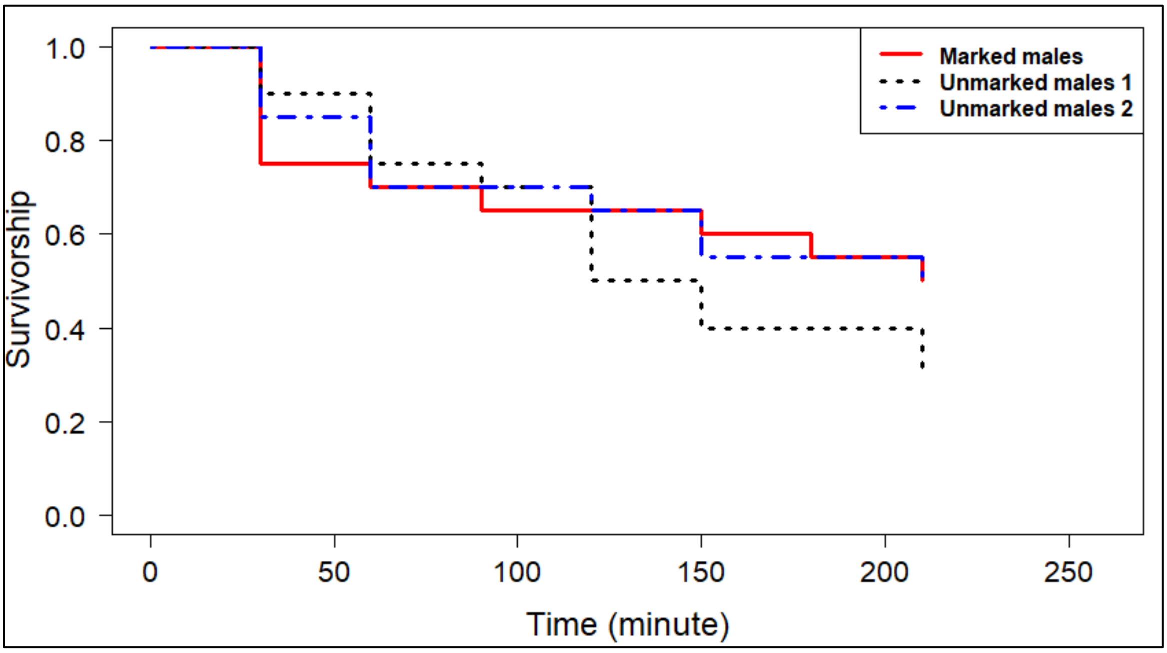

Supplementary Fig. S1 Dynamic of *Phyllocrania paradoxa* predation on marked and unmarked male *Aedes albopictus*

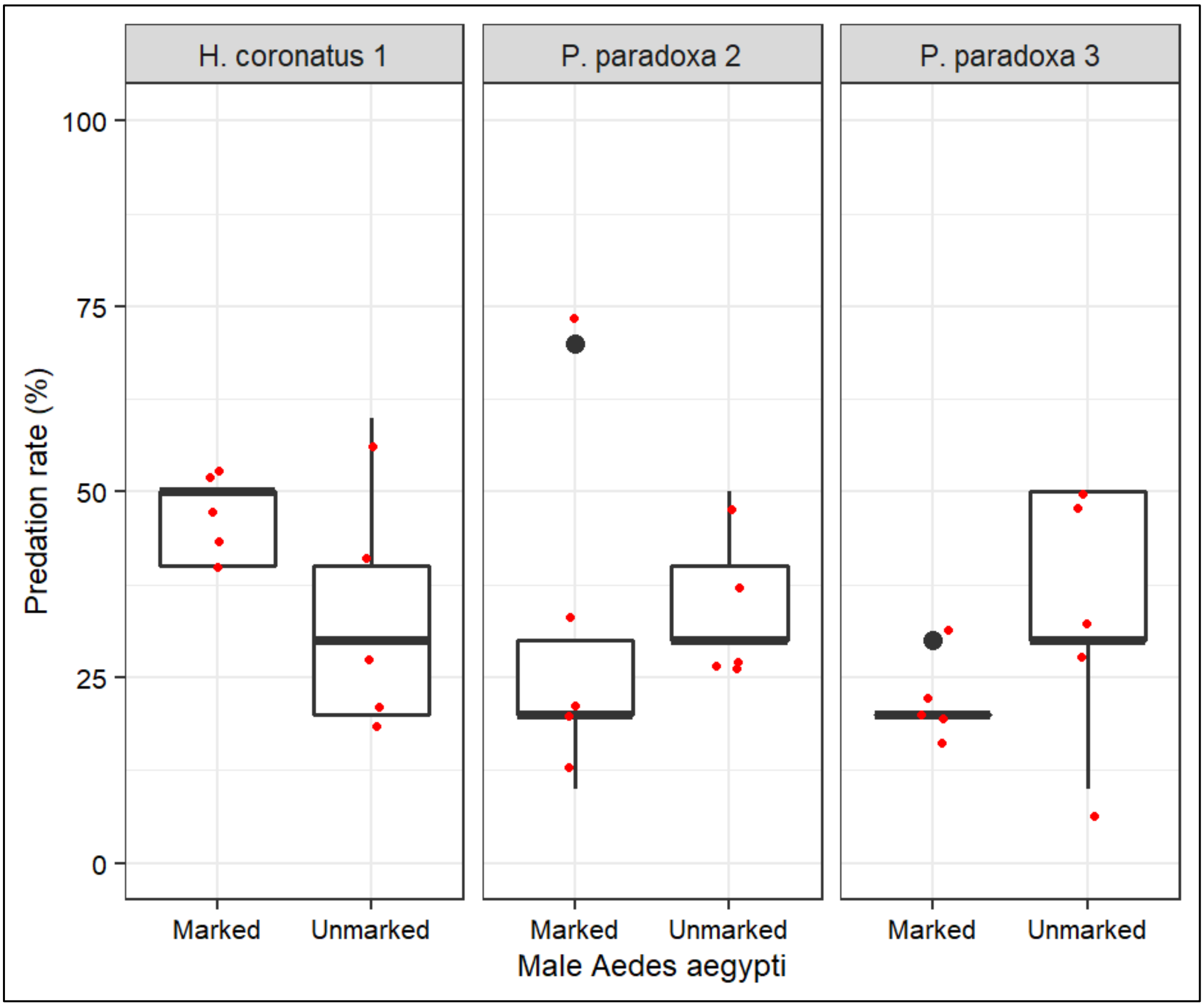

Supplementary Fig. S2 Mantis predation rate on marked and unmarked male *Aedes aegypti*

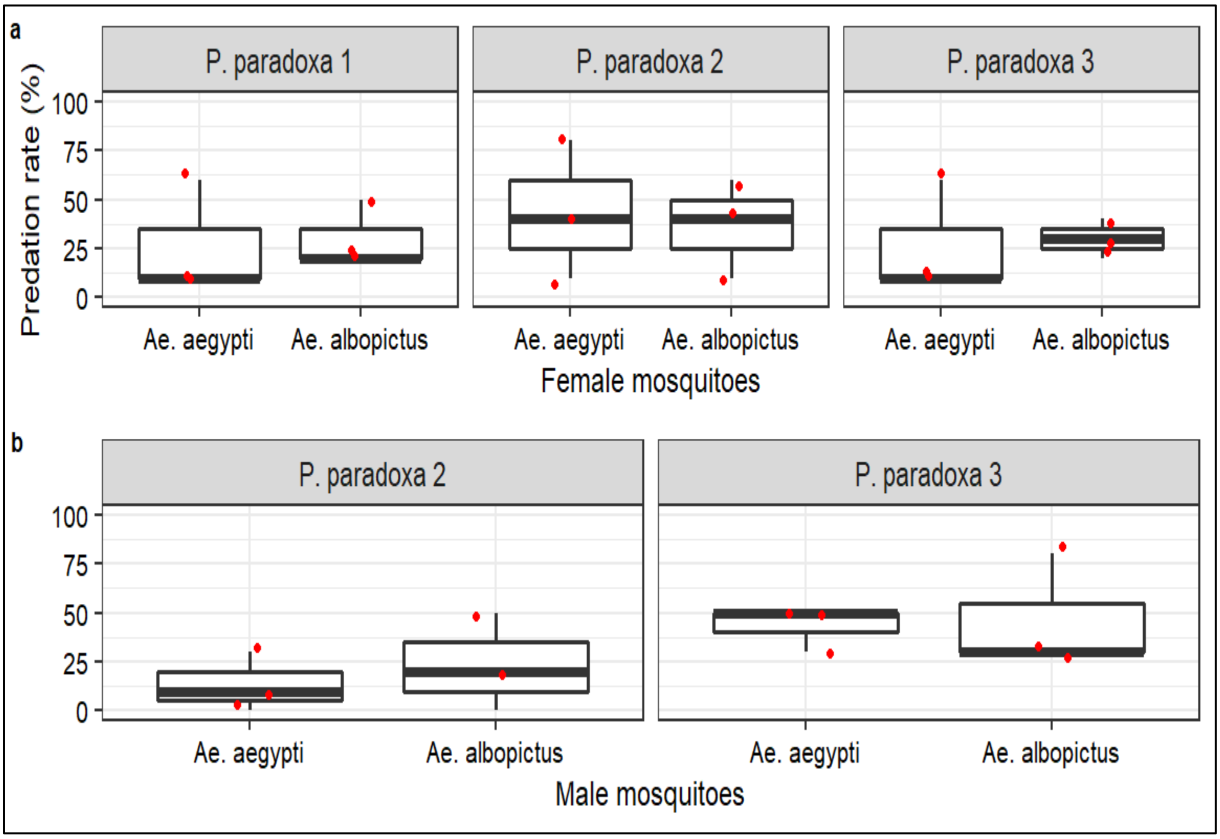

Supplementary Fig. S3 Mantis predation preference between mosquito species

a: male *Aedes albopictus versus* male *Aedes aegypti* b: female *Aedes albopictus versus* female *Aedes aegypti*

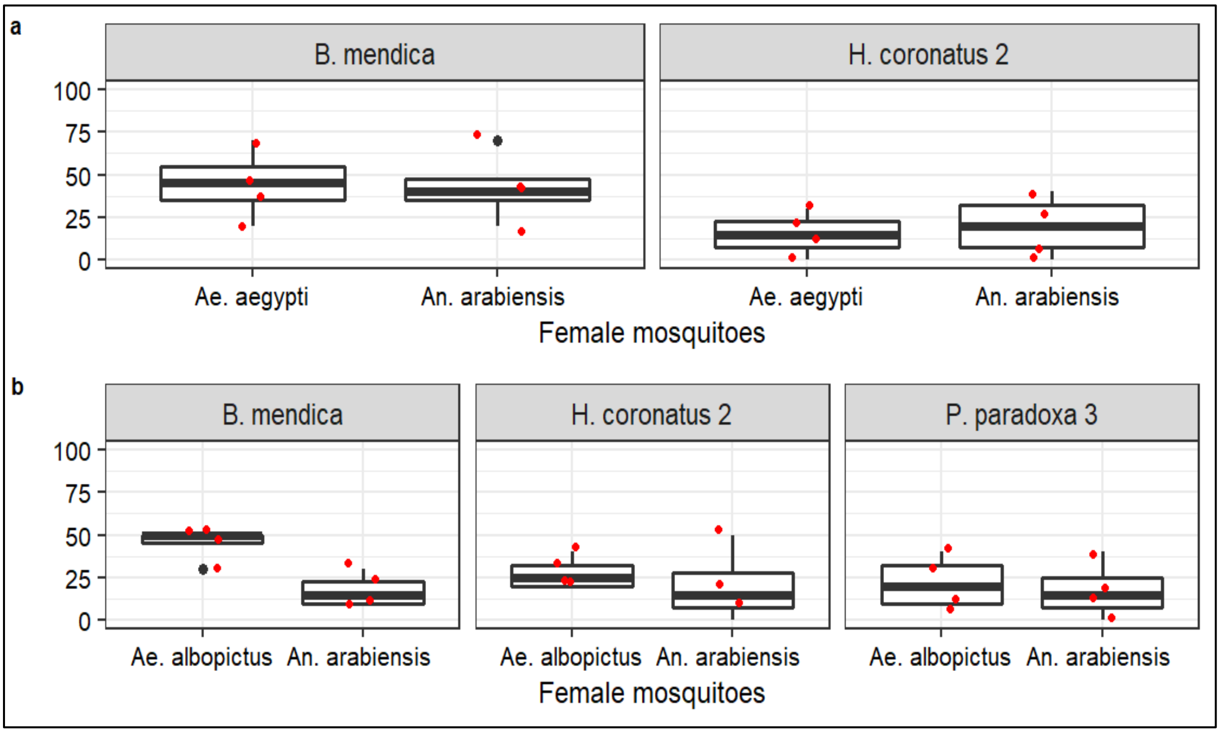

Supplementary Fig. S4 Mantis predation preference between mosquito species

a: female *Aedes aegypti versus* female *Anopheles arabiensis; b:* female *Aedes albopictus versus* female *Anopheles arabiensis*

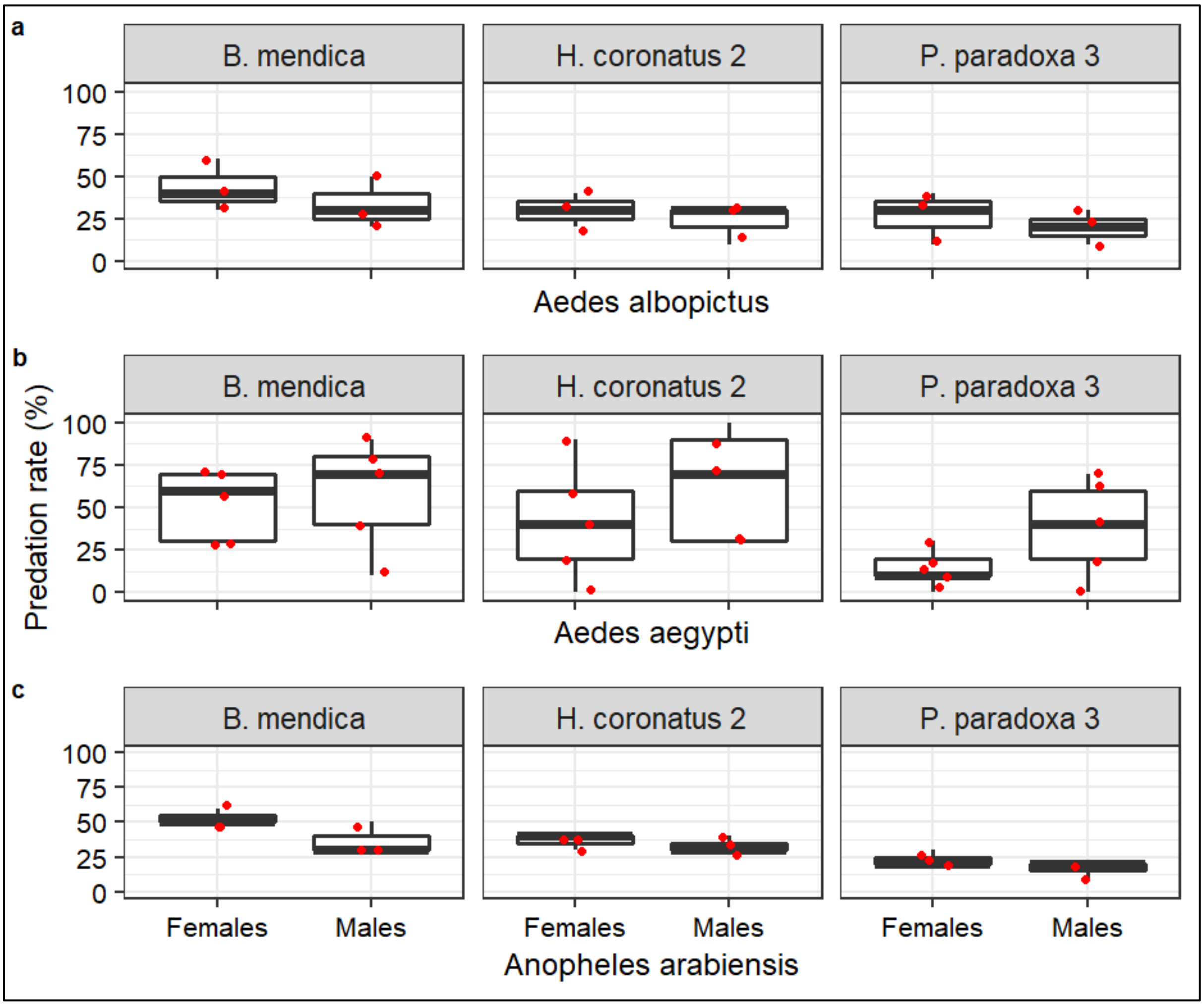

Supplementary Fig. S5 Mantis predation preference between male and female mosquitoes

a: *Aedes albopictus;* b: *Aedes aegypti;* c: *Anopheles arabiensis*

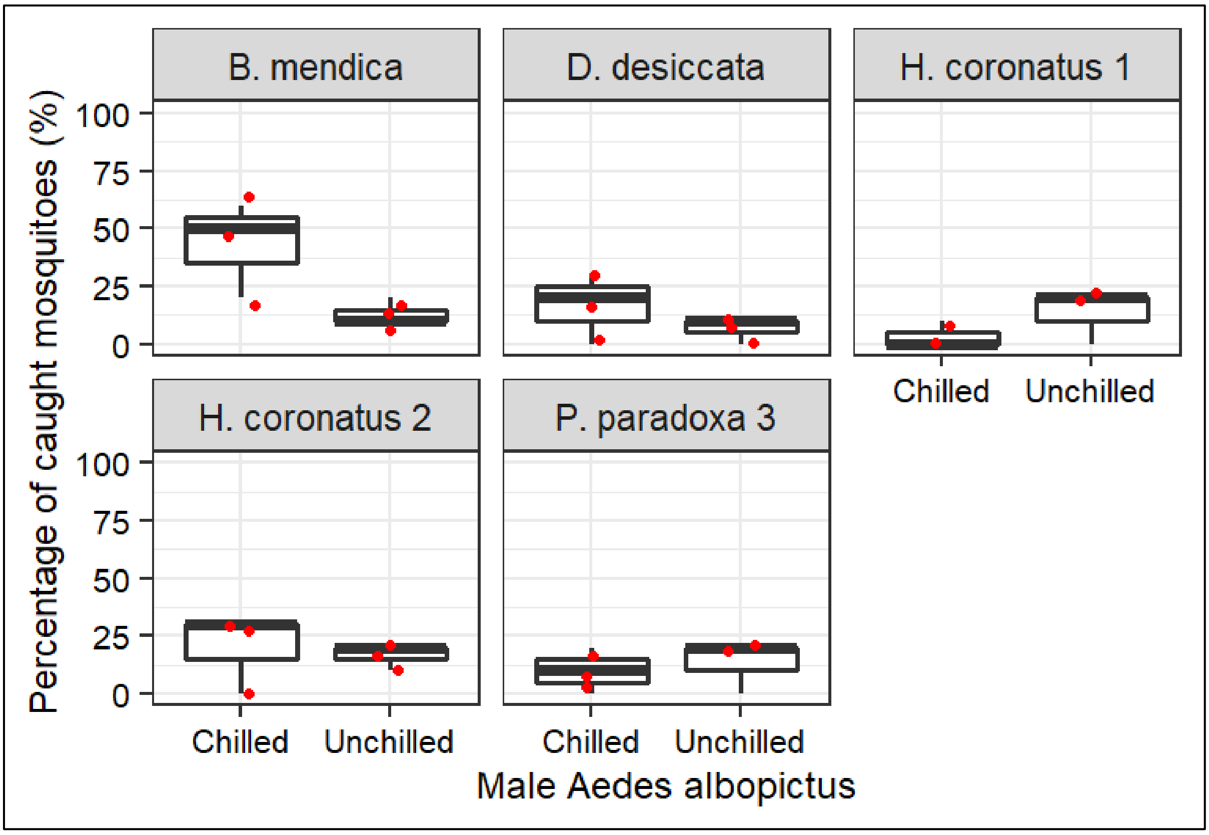

Supplementary Fig. S6 Vulnerability of chilled versus non chilled male *Aedes albopictus* to mantis predation

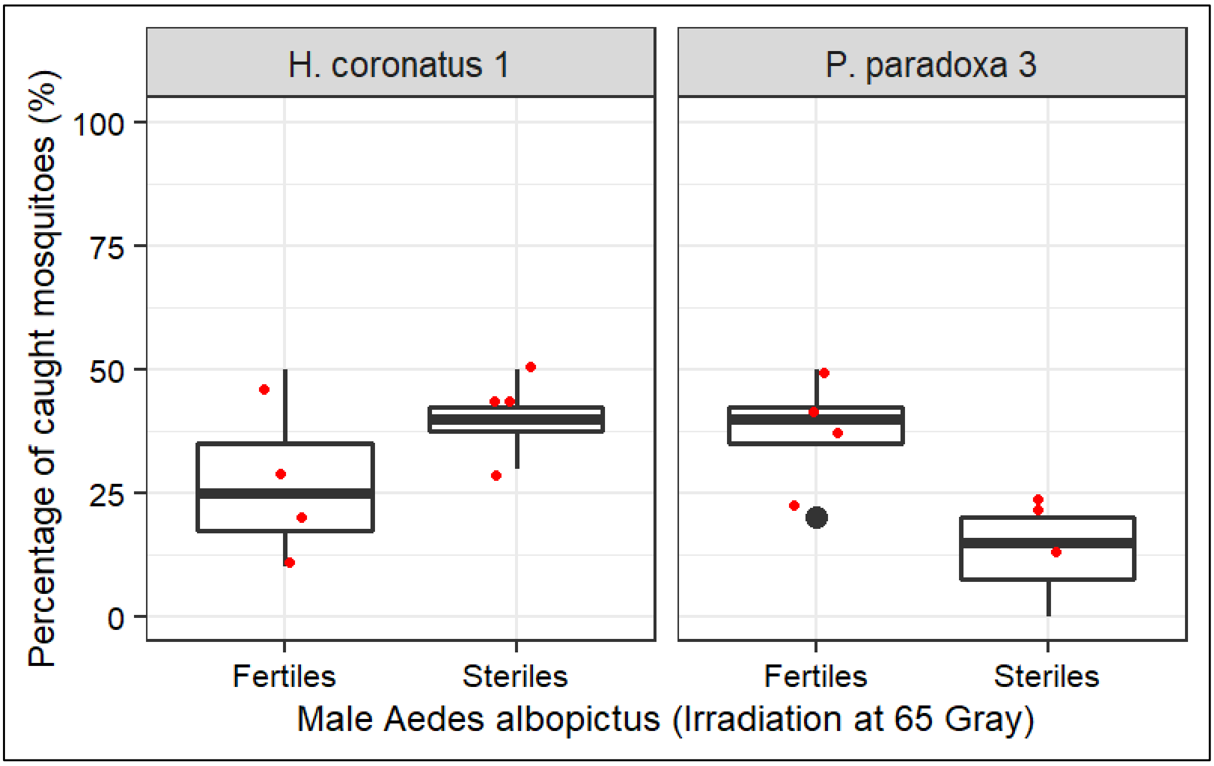

Supplementary Fig. S7 Vulnerability of fertile versus sterile male *Aedes albopictus* (irradiated at 65 Gy) to mantis predation

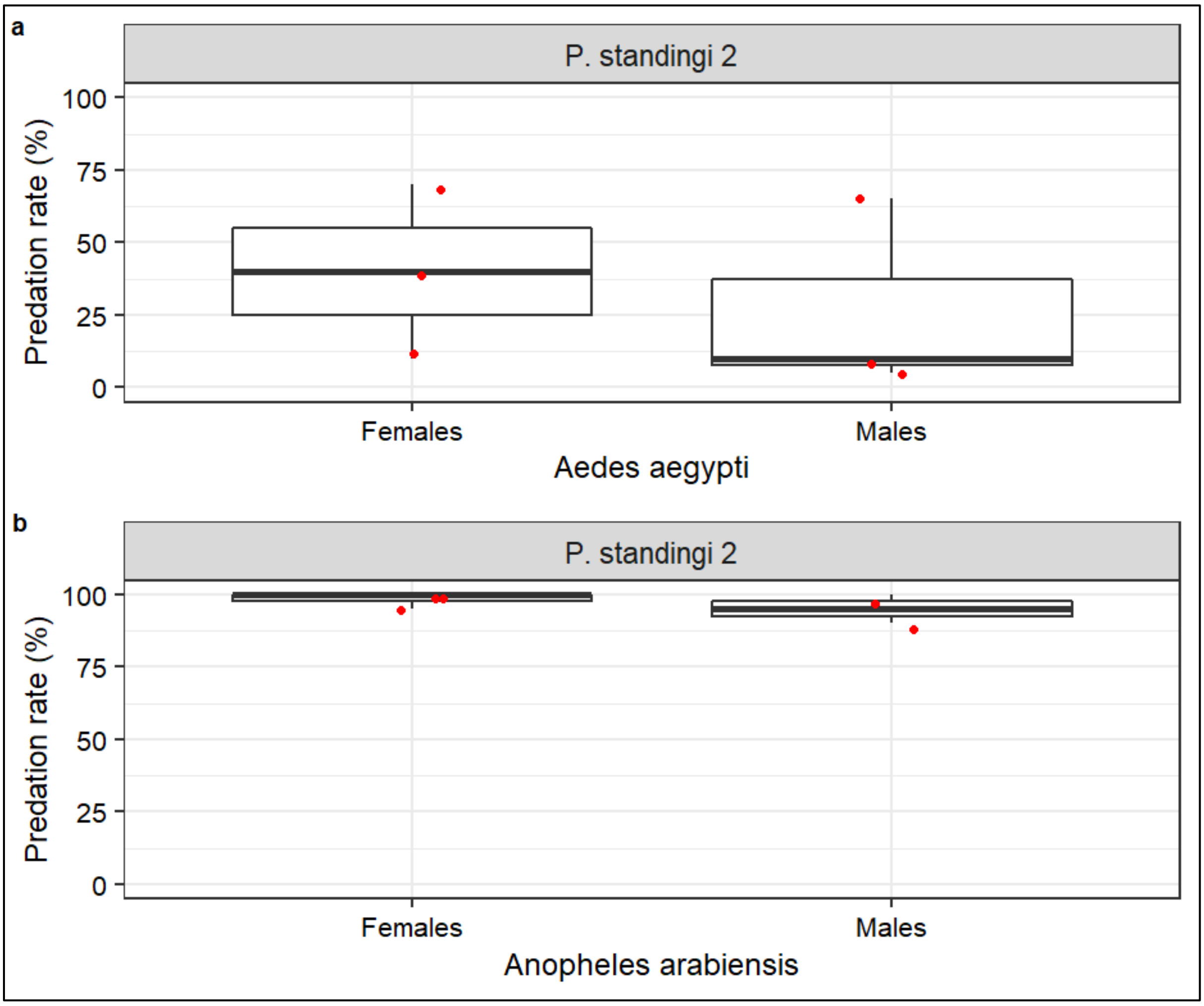

Supplementary Fig. S8 *Phelsuma standingi* predation preference for male or female mosquitoes

a: *Aedes aegypti;* b: *Anopheles arabiensis*

Supporting Tables

Supplementary Table S1 Pairwise comparisons of *Phyllocrania paradoxa* individuals' *predation* propensity on *Aedes aegypti* mosquitoes

contrast	ratio	SE	df	null	z.ratio	p.value
P. paradoxa 1 / P. paradoxa 2	0.923	0.310	Inf	1	-0.240	0.9688
P. paradoxa 1 / P. paradoxa 3	0.671	0.223	Inf	1	-1.202	0.4518
P. paradoxa 2 / P. paradoxa 3	0.727	0.238	Inf	1	-0.975	0.593

Supplementary Table S2 Pairwise comparisons of the vulnerability of marked and unmarked *Aedes albopictus* mosquitoes to *Phyllocrania paradoxa* predation

contrast	ratio	SE	df	null	z.ratio	p.value
Marked / Unmarked 1	0.676	0.281	Inf	1	-0.942	0.6135
Marked / Unmarked 2	1.028	0.460	Inf	1	0.062	0.9979
Unmarked 1 / Unmarked 2	1.521	0.631	Inf	1	1.010	0.5702

Supplementary Table S3 Relative vulnerability of mosquito species to mantises' predation

Test		Chisq	Df	Pr(>Chisq)
Female *Ae. abopictus* versus female *Ae. agypti*	Mantis	2.5659	2	0.2772
	Mosquito species	0.0000	1	0.9991
	Mantis:Mosquito species	0.4615	2	0.7940
Male *Ae abopictus* versus Male *Ae agypti* males	Mantis	9.1412	1	**0.0025**
	Mosquitoes	0.6449	1	0.4219
	Mantis:Mosquito species	0.4026	1	0.5257
Female *An arabiensis* versus female *Ae agypti*	Mantis	12.1006	1	0.0005
	Mosquitoes	0.0308	1	0.8607
	Mantis:Mosquitoes	0.3643	1	0.5461
Female *An arabiensis* versus female *Ae albopictus*	Mantis	2.8374	2	0.24203
	Mosquitoes	5.2805	1	**0.02157**
	Mantis:Mosquitoes	2.2943	2	0.31754

Supplementary Table S4 Pairwise comparisons of mantises' predation propensity on *Anopheles arabiensis* (males *vs* females*)* mosquitoes

contrast	odds.ratio	SE	df	null	z.ratio	p.value
B. mendica / *H. coronatus* 2	1.52	0.570	Inf	1	1.116	0.5043
B. mendica / *P. paradoxa* 3	3.27	1.355	Inf	1	2.863	**0.0117**
H. coronatus 2 / *P. paradoxa* 3	2.15	0.907	Inf	1	1.822	0.1625

SFSupplementary Table S5 Pairwise comparisons of mantises' predation propensity on chilled versus unchilled males *Aedes albopictus*

**contrast**	**odds.ratio**	**SE**	**df**	**null**	**z.ratio**	**p.value**
B. mendica / D. desiccata	3.077	1.532	Inf	1	2.257	0.1592
B. mendica / H. coronatus 1	4.494	2.478	Inf	1	2.725	**0.0503**
B. mendica / H. coronatus 2	1.791	0.796	Inf	1	1.310	0.6853
B. mendica / P. paradoxa 3	3.077	1.532	Inf	1	2.257	0.1592
D. desiccata / H. coronatus 1	1.461	0.903	Inf	1	0.613	0.9731
D. desiccata / H. coronatus 2	0.582	0.306	Inf	1	-1.030	0.8416
D. desiccata / P. paradoxa 3	1.000	0.571	Inf	1	0.000	1.0000
H. coronatus 1 / H. coronatus 2	0.398	0.230	Inf	1	-1.597	0.4995
H. coronatus 1 / P. paradoxa 3	0.685	0.423	Inf	1	-0.613	0.9731
H. coronatus 2 / P. paradoxa 3	1.718	0.903	Inf	1	1.030	0.8416

Supplementary Table S6 Relative vulnerability of irradiated (at 70 Gy) versus unirradiated males *Aedes aegypti* to mantises' predation

	Chisq	Df	Pr(>Chisq)
Mantis	26.6476	4	2.342e-05
Mosquito group	0.0036	1	0.95184
Mantis:Mosquito group	9.6753	4	**0.04627**

Supplementary Table S7 Pairwise comparisons of mantises' predation propensity on unirradiated versus irradiated males *Aedes aegypti*

**Mantis invidividual**	**contrast**	**odds ratio**	**SE**	**df**	**null**	**z.ratio**	**p.value**
B. mendica	Fertile / Sterile	1.000	0.526	Inf	1	<0.001	1.0000
D. desiccata	Fertile / Sterile	4.571	3.303	Inf	1	2.103	**0.0355**
H. coronatus 1	Fertile / Sterile	1.444	0.717	Inf	1	0.741	0.4587
H. coronatus 2	Fertile / Sterile	0.546	0.351	Inf	1	-0.941	0.3469
P. paradoxa 3	Fertile / Sterile	0.296	0.184	Inf	1	-1.961	**0.0499**
**Mosquito group**	**contrast**	**Odds ratio**	**SE**	**df**	**null**	**z.ratio**	**p.value**
**Fertile**	B. mendica / D. desiccata	2.657	1.431	Inf	1	1.814	0.3653
	B. mendica / H. coronatus 1	2.95	1.531	Inf	1	2.084	0.2268
	B. mendica / H. coronatus 2	6.717	4.139	Inf	1	3.091	**0.017**
	B. mendica / P. paradoxa 3	16.435	10.84	Inf	1	4.245	**0.0002**
	D. desiccata / H. coronatus 1	1.11	0.59	Inf	1	0.197	0.9997
	D. desiccata / H. coronatus 2	2.528	1.585	Inf	1	1.48	0.5758
	D. desiccata / P. paradoxa 3	6.186	4.139	Inf	1	2.723	**0.0506**
	H. coronatus 1 / H. coronatus 2	2.277	1.388	Inf	1	1.35	0.6599
	H. coronatus 1 / P. paradoxa 3	5.571	3.413	Inf	1	2.804	0.0405
	H. coronatus 2 / P. paradoxa 3	2.447	1.792	Inf	1	1.222	0.7387
**Sterile**	B. mendica / D. desiccata	12.144	8.665	Inf	1	3.499	**0.0043**
	B. mendica / H. coronatus 1	4.261	2.281	Inf	1	2.707	**0.0529**
	B. mendica / H. coronatus 2	3.67	2.045	Inf	1	2.333	0.1344
	B. mendica / P. paradoxa 3	4.859	2.638	Inf	1	2.912	**0.0296**
	D. desiccata / H. coronatus 1	0.351	0.253	Inf	1	-1.455	0.5917
	D. desiccata / H. coronatus 2	0.302	0.223	Inf	1	-1.625	0.4813
	D. desiccata / P. paradoxa 3	0.4	0.29	Inf	1	-1.263	0.714
	H. coronatus 1 / H. coronatus 2	0.861	0.487	Inf	1	-0.264	0.9989
	H. coronatus 1 / P. paradoxa 3	1.14	0.584	Inf	1	0.257	0.999
	H. coronatus 2 / P. paradoxa 3	1.324	0.758	Inf	1	0.49	0.9883

Supplementary Table S8 Relative vulnerability of irradiated (at 65 Gy) versus unirradiated males *Aedes albopictus* to mantises' predation

	**Chisq**	**Df**	**Pr(>Chisq)**
Mantis	0.9626	1	0.32653
Mosquitoes	0.4541	1	0.5004
Mantis:Mosquitoes	7.0781	1	**0.0078**
