# Supplementary material for: Adult mosquito predation and potential impact on the sterile insect technique
Source: Sci Rep. 2022 Feb 15;12:2561. doi: 10.1038/s41598-022-06565-1 (PMC8847352; doi:10.1038/s41598-022-06565-1)
Supplement: Supplementary file 2 — Supplementary Legends. [file 41598_2022_6565_MOESM2_ESM.docx]

**Video captions**

**Video 1:** *Phyllocrania paradoxa* preying on *Aedes* mosquitoes using sit-and-wait hunting mode (Credit: BIMBILE SOMDA NS)

**Video 2:** *Phyllocrania paradoxa* preying on *Aedes* mosquitoes using active hunting mode (Credit: BIMBILE SOMDA NS)

**Video 3:** *Phelsuma standingi* preying on *Aedes* mosquitoes using active hunting mode (Credit: BIMBILE SOMDA NS)
